# Post-traumatic high-flow priapism treated by endovascular embolization using N-butyl-cyanoacrylate

**DOI:** 10.2478/v10019-010-0024-x

**Published:** 2010-05-24

**Authors:** Marko Rados, Vice Sunjara, Ivica Sjekavica, Ranka Stern Padovan

**Affiliations:** University Department of Diagnostic and Interventional radiology, University Hospital Center Zagreb, Zagreb, Croatia

**Keywords:** priapism, endovascular embolization, angiography, Doppler duplex ultrasonography, MRI angiography

## Abstract

**Background:**

Priapism, persistent erection without arousal, can be classified into low-flow (venous or ischemic) and high-flow (arterial or non-ischemic). The diagnosis of high-flow priapism can be confirmed by colour Doppler and arteriography and it is usually treated by the endovascular embolization.

**Case report:**

We present a case of a 20-year-old man with a post-traumatic high-flow priapism as a result of the previous perineal trauma. After a period of watchful waiting and an unsuccessful attempt at endovascular embolization using the resorptive gelatinous foam he was successfully treated by the endovascular embolization using N-butyl-cyanoacrylate.

**Conclusions:**

High-flow priapism can be successfully treated by the endovascular embolization, but the optimal choice of the embolization agent and a careful technique is essential.

## Introduction

Priapism is a relatively rare condition characterized by the persistent erection in the absence of sexual arousal. There are two main subtypes: the more common ischemic, or low-flow, characterized by the impaired outflow from the corpora cavernosa, and non-ischemic, or high-flow, most often caused by trauma, characterized by the formation of arteriocavernous fistulas and increased inflow of blood to the corpora cavernosa. While the painful low-flow priapism and the associated decreased oxygenation of cavernous tissue can quickly lead to a cavernous fibrosis and permanent damage to penile tissues and is, therefore, an urological emergency, high-flow priapism is often painless and can persist for months or years, in most cases without a permanent damage of penile tissues, but sometimes with the reduced potency.^1^

The diagnosis of high-flow priapism can be confirmed by colour Doppler^2^, which can also be used to characterize the number and location of arteriocavernous fistulas and concomitant arterial pathology such as pseudoaneurysms. Colour Doppler is also useful in the follow-up, avoiding the repeated angiography with its risks and the ionizing radiation dose, although the MR angiography is usually necessarily to evaluate the effect of the radiological invasive interventional procedures.^3^

There are many treatment options in high-flow priapism: those mentioned most often are watchful waiting^4^, Doppler-guided compression^5^, endovascular highly selective embolization and surgery. Because the more aggressive treatment methods are associated with a small but significant rate of the permanent erectile dysfunction, an initial watchful waiting period is commonly indicated. The surgery in high-flow priapism usually consists of the ligation of a cavernous artery or its branch and is reported to have the highest permanent erectile dysfunction rate, thus it is usually the last treatment option.

## Case report

**FIGURE 1:**
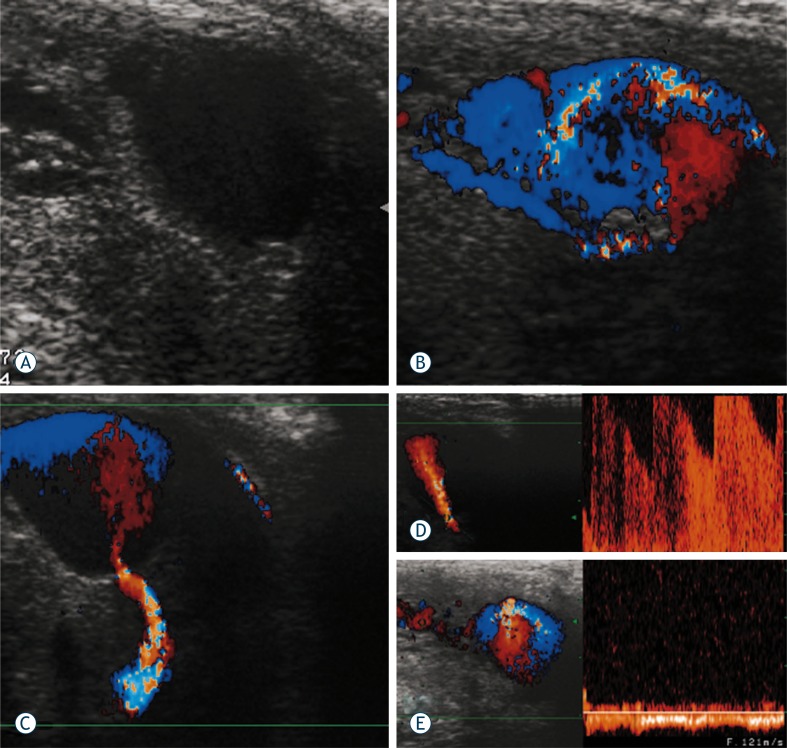
**A.** Gray-scale ultrasound depicts anehoic region, within corpus cavernosum. **B.** Colour Doppler ultrasound with multiple colour signals. **C.** Doppler sonogram of cavernous artery which fills the pseudoaneurysm. **D.** Pulsed Doppler analysis with aliasing phenomena due to turbulent high-velocity flow in the cavernous artery. **E.** Venous drainage in the *corpora cavenosa* on Doppler sonogram.

A 20-year old patient presented with priapism caused by previous perineal trauma. Gray-scale ultrasound depicted anehoic region, 14.7 x 12.7 mm in size, within corpus cavernosum (Figure 1A) Colour Doppler ultrasound showed multiple colour signals due to the extravasation of blood (Figure 1B). The pulsed Doppler analysis confirmed typical to-fro signals into suspected cavernoma and a high velocity flow in the cavernous artery which fills the pseudoaneurysm (Figure 1C and 1D). Venous drainage in corpora cavenosa was also found on Doppler examination (Figure 1E)

**FIGURE 2:**
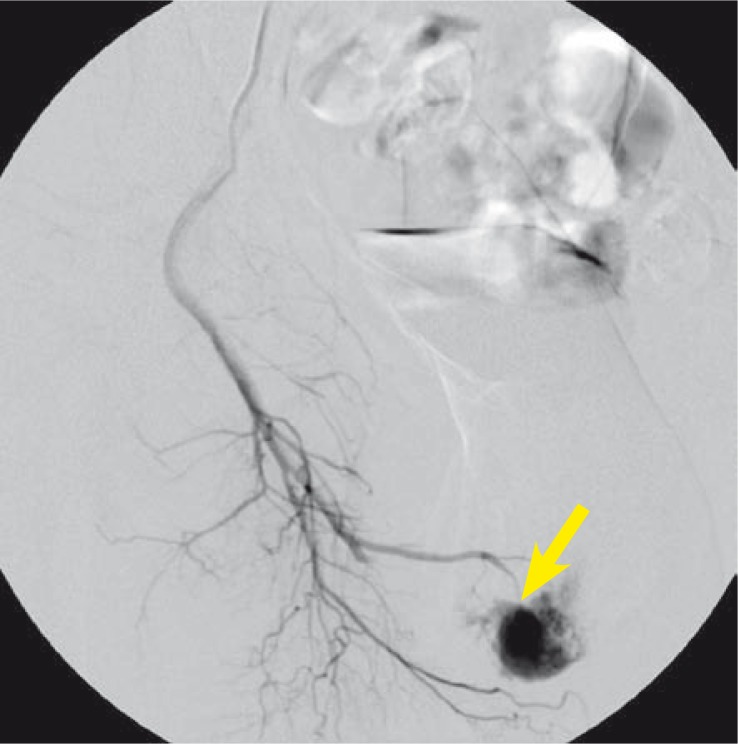
Selective angiography before embolization shows the arteriocavernous fistula (marked with an arrow).

The initial arteriography confirmed a pseudoaneurysm of the right cavernous artery with an arteriocavernous fistula (Figure 2). A smaller arteriocavernous fistula was also present on the left cavernous artery. The communication of the left and right internal pudendal artery was noted, with the blood from the left pudendal artery flowing to the right and contributing to the filling of the pseudoaneurysm on the right.

**FIGURE 3:**
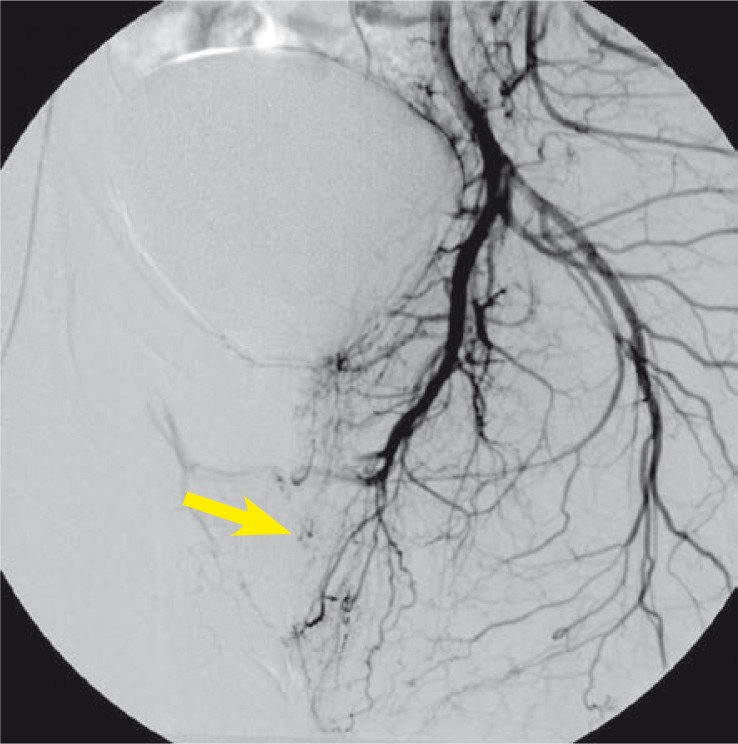
Selective angiography after the second embolization shows the occlusion of arteriocavernous fistula (filling artery marked with an arrow).

**FIGURE 4:**
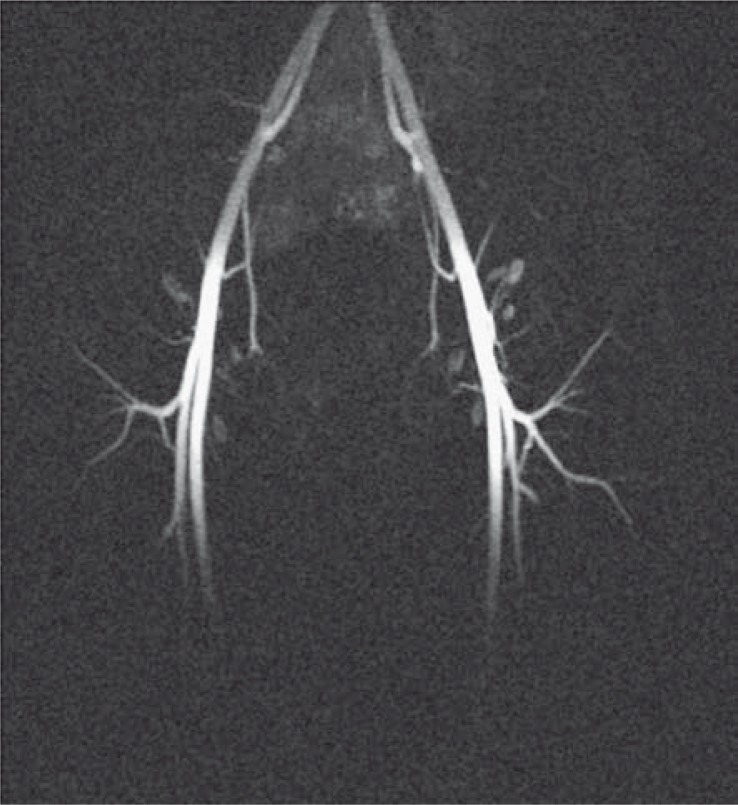
Contrast-enhanced MR angiography, late follow-up: a maximum intensity projection of the pelvic vessels in the late arterial phase shows no abnormalities.

After a six-month period of watchful waiting priapism did not resolve spontaneously and a more aggressive approach was decided upon with an attempt at highly selective embolization of the fistulas of both cavernous arteries using the resorptive gelatinous foam. One-month follow-up showed a recurrence of the right-sided fistula necessitating another embolization procedure during which the superselective catheterization was performed and a microcatheter was inserted into the pseudoaneurysm on the right cavernous artery. The embolization agent used was N-butyl-cyanoacrylate (Glubran II, GEM S.r.l., Viareggio, Italy). Two-month follow-up showed the closure of arteriocavernous fistulas with the persistence of pseudoaneurym on the right that had morphed into a small cavernoma, which was embolized using additional N-butyl-cyanoacrylate. The end-result was a complete occlusion of the fistula (Figure 3). Priapism was successfully resolved and the patient remained symptom-free and regained the erectile function. Contrast-enhanced MR angiography follow-up at 6 months showed no recurrence of the fistula (Figure 4).

## Discussion

The endovascular selective embolization of the pathological arteriocavernous communication is firmly established as the invasive treatment of choice in high-flow priapism.^6^,^7^ It is commonly performed using microcatheters and a range of embolization materials: autologous clots, gelatinous foam, endovascular coils^8^ or N-butyl-cyanoacrylate.^9^,^10^ Autologous clots and gelatinous foam are often preferred because of their spontaneous degradation and a reportedly lower risk of the permanent erectile dysfunction, but could have a greater recurrence rate.

After our first unsuccessful attempt at embolization using resorptible embolization materials we switched to N-butyl-cyanoacrylate (Glubran II) which could provide faster and more efficient occlusion of the fistula. The cyanoacrylate embolization is permanent and carries a higher risk of ischemia of the vessel in question, and it consequently requires a better embolization technique and more experienced interventionists capable of introducing the catheter and the embolization material directly into the site of the fistula. In our case the treatment was facilitated by the fistula being positioned on a pseudoaneurysm of the cavernous artery. Even though the fistula was occluded after the first embolization session with N-butyl-cyanoacrylate we elected to perform an additional session to obliterate the residual cavernoma in order to prevent a possible recanalization of the fistula and recurrence.

Our case showed that embolization using N-butyl-cyanoacrylate (Glubran II) could be used as a second-line treatment in patients with recurrence after the first embolization attempt with resorptible materials.
